# Bis(μ-naphthalene-1,8-dicarboxyl­ato-κ^2^ *O* ^1^:*O* ^8^)bis­[aqua­bis­(*N*,*N*′-dimethyl­formamide-κ*O*)copper(II)]

**DOI:** 10.1107/S1600536810028497

**Published:** 2010-07-24

**Authors:** Jun-Dan Fu, Chun-Yan Zhang, Qing-Yu Shi, Yi-Hang Wen

**Affiliations:** a Zhejiang Key Laboratory for Reactive Chemistry on Solid Surfaces, Institute of Physical Chemistry, Zhejiang Normal University, Jinhua, Zhejiang 321004, People’s Republic of China

## Abstract

In the centrosymmetric dinuclear title complex, [Cu_2_(C_12_H_6_O_4_)_2_(C_3_H_7_NO)_4_(H_2_O)_2_], the coordination environment of each Cu(II) atom displays a distorted CuO_5_ square-pyramidal geometry, which is formed by two carboxyl­ate O atoms of two μ-1,8-nap ligands (1,8-nap is naphthalene-1,8-dicarboxyl­ate), two O atoms of two DMF (DMF is *N*,*N*′-dimethyl­formamide) and one coordinated water mol­ecule. The Cu—O distances involving the four O atoms in the square plane are in the range 1.9501 (11)–1.9677 (11) Å, with the Cu atom lying nearly in the plane [deviation = 0.0726 (2) Å]. The axial O atom occupies the peak position with a Cu—O distance of 2.885 (12) Å, which is significantly longer than the rest of the Cu—O distances. Each 1,8-nap ligand acts as bridge, linking two Cu^II^ atoms into a dinuclear structure. Inter­molecular O—H⋯O and C—H⋯O hydrogen-bonding inter­actions consolidate the structure.

## Related literature

For the coordination modes of the 1,8-nap ligand, see: Wen *et al.* (2007 ▶, 2008 ▶). For related complexes, see: Abourahma *et al.* (2002 ▶); Bencini *et al.* (2003 ▶); Fokin *et al.* (2004 ▶); Sun *et al.* (2009 ▶).
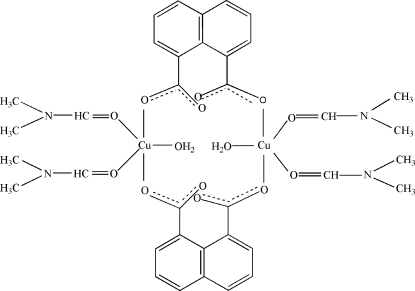


## Experimental

### 

#### Crystal data

[Cu_2_(C_12_H_6_O_4_)_2_(C_3_H_7_NO)_4_(H_2_O)_2_]
*M* *_r_* = 883.83
Monoclinic, 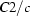

*a* = 17.7078 (4) Å
*b* = 9.9025 (1) Å
*c* = 23.0393 (5) Å
β = 102.249 (2)°
*V* = 3948.00 (13) Å^3^
*Z* = 4
Mo *K*α radiation
μ = 1.15 mm^−1^
*T* = 296 K
0.40 × 0.26 × 0.13 mm

#### Data collection

Bruker APEXII area-detector diffractometer
Absorption correction: multi-scan (*SADABS*; Sheldrick, 1996 ▶) *T*_min_ = 0.71, *T*_max_ = 0.86
29673 measured reflections
4625 independent reflections
4076 reflections with *I* > 2σ(*I*)
*R*_int_ = 0.025

#### Refinement

*R*[*F* ^2^ > 2σ(*F* ^2^)] = 0.027
*wR*(*F* ^2^) = 0.076
*S* = 1.04
4625 reflections
259 parameters
5 restraints
H atoms treated by a mixture of independent and constrained refinement
Δρ_max_ = 0.28 e Å^−3^
Δρ_min_ = −0.27 e Å^−3^

### 

Data collection: *APEX2* (Bruker, 2006 ▶); cell refinement: *SAINT* (Bruker, 2006 ▶); data reduction: *SAINT*; program(s) used to solve structure: *SHELXS97* (Sheldrick, 2008 ▶); program(s) used to refine structure: *SHELXL97* (Sheldrick, 2008 ▶); molecular graphics: *SHELXTL* (Sheldrick, 2008 ▶); software used to prepare material for publication: *SHELXTL*.

## Supplementary Material

Crystal structure: contains datablocks I, global. DOI: 10.1107/S1600536810028497/pv2300sup1.cif

Structure factors: contains datablocks I. DOI: 10.1107/S1600536810028497/pv2300Isup2.hkl

Additional supplementary materials:  crystallographic information; 3D view; checkCIF report

## Figures and Tables

**Table 1:** Hydrogen-bond geometry (Å, °)

*D*—H⋯*A*	*D*—H	H⋯*A*	*D*⋯*A*	*D*—H⋯*A*
O1*W*—H1*WA*⋯O5	0.82 (2)	1.82 (2)	2.642 (2)	178 (2)
O1*W*—H1*WB*⋯O4^i^	0.80 (1)	1.82 (2)	2.623 (2)	175 (2)
C3—H3*A*⋯O1^ii^	0.93	2.49	3.396 (3)	164
C13—H13*A*⋯O3	0.93	2.59	3.140 (2)	119
C17—H17*A*⋯O6^iii^	0.96	2.51	3.424 (3)	159

Symmetry codes: (i) 
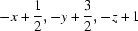
; (ii) 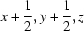; (iii) 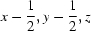.

**Fig. 1.**
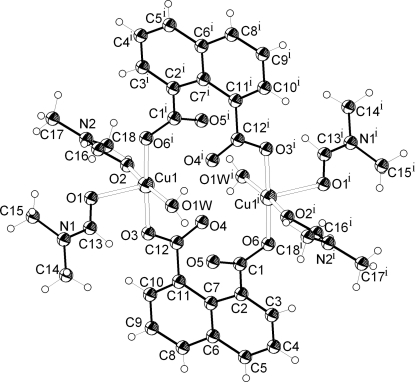
Perspective view of the structure of (I), showing the atom-numbering scheme. Displacement ellipsoids are drawn at the 30% probability level. [Symmetry codes: (i)-x + 1/2,-y + 3/2,-z + 1]

**Fig. 2.**
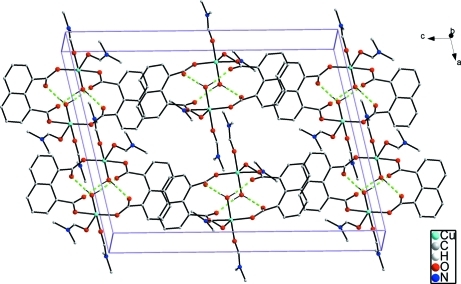
A packing diagram of (I) with the hydrogen-bonding interaction depicted by dash lines.

## supplementary crystallographic information

### Comment

It is well-known that appropriate metal and ligand are the two keys for design and construction of metal-organic frameworks. Here we choose 1,8-nap ligand (1,8-nap = naphthalene-1,8-dicarboxylate), due to its unique ability to form stable chelates in diverse coordination modes such as bidentate, meridian and bridging; which have been demonstrated in our previous work (Wen *et al.*, 2008; Wen *et al.*, 2007). Moreover, we select the copper to provide a set of well defined coordination geometry. As a result, we have prepared the title complex, Cu_2_(1,8-nap)_2_(DMF)_4_(H_2_O)_2_, (I), a new dinuclear Cu^II^ compound based on 1,8-nap ligand.

A perspective view of the molecular structure of (I) is presented in Fig. 1. The coordination environment of each Cu atom displays a distorted CuO_5_ square pyramidal coordination geometry, which is formed from two carboxylate oxygen atoms of two µ_2_-1,8-nap ligands, two oxygen atoms of two DMF and one coordinated water molecule; similar to some comlexes reported earlier (Abourahma *et al.*, 2002; Bencini *et al.*, 2003; Fokin *et al.*, 2004; Sun *et al.*, 2009). Four oxygen atoms O2-O3^i^-O1W-O6 form a square plane (Cu—O distances in ranging 1.9501 (11) - 1.9677 (11) Å), and the Cu1 atom lies in the plane (deviation 0.0726 (2) Å). The fifth oxygen atom O1 is on the peak of square pyramid, and Cu—O distance is 2.885 (12) Å, which is significantly longer than the rest of the Cu—O distances. Both carboxylate groups of the 1,8-nap ligand are deprotonated, and adopt a monodentate coordination mode. As a result, the whole 1,8-nap ligand acts as µ_2_-bridge linking two Cu^II^ atoms to form a sixteen-atoms ring. There are intramolecular hydrogen bonds between uncoordinated O atoms of 1,8-nap ligands and water molecules, O1W···O5, O1W···O4^i^(details are given in Tab. 1). In addition, weak interactions of the type C—H···O are also present. Such hydrogen-bonding interactions consolidate the dinuclear structure, as depicted in Fig. 2.

### Experimental

A mixture of naphthalene-1,8-dicarboxylate anhydride (0.1981 g, 1 mmol), CuCl_2_.2H_2_O (0.085 g, 0.5 mmol) and Na_2_CO_3_(0.053 g, 0.5 mmol) was dissolved in a mixed solution of DMF-H_2_O (1:2 *v*/*v*, 25 ml) and stirred at 343 K for 2 h. The filtrate was allowed to stand at ambient temperature. Well formed blue crystals suitable for X-ray analysis were obtained after two months (yield 45%, based on Cu).

### Refinement

H atoms bonded to C atoms were positioned geometrically and included in the refinement in the riding-model approximation [C—H = 0.93 Å and *U*_iso_(H) = 1.2*U*_eq_(C)] and methyl groups were allowed to rotate to fit the electron density [C—H = 0.96 Å and *U*_iso_(H) = 1.5*U*_eq_(C)]. Water H atoms were located and refined with distance restraints of O—H = 0.85 (2) Å and H···H = 1.35 (2) Å, with displacement parameters set at 1.5*U*_eq_(O).

### Crystal data

**Table:**

[Cu_2_(C_12_H_6_O_4_)_2_(C_3_H_7_NO)_4_(H_2_O)_2_]	*F*(000) = 1832
*M**_r_* = 883.83	*D*_x_ = 1.487 Mg m^−^^3^
Monoclinic, *C*2/*c*	Mo *K*α radiation, λ = 0.71073 Å
Hall symbol: -C 2yc	Cell parameters from 9928 reflections
*a* = 17.7078 (4) Å	θ = 2.4–27.7°
*b* = 9.9025 (1) Å	µ = 1.15 mm^−^^1^
*c* = 23.0393 (5) Å	*T* = 296 K
β = 102.249 (2)°	Block, blue
*V* = 3948.00 (13) Å^3^	0.40 × 0.26 × 0.13 mm
*Z* = 4	

### Data collection

**Table:**

Bruker APEXII area-detector diffractometer	4625 independent reflections
Radiation source: fine-focus sealed tube	4076 reflections with *I* > 2σ(*I*)
graphite	*R*_int_ = 0.025
ω scans	θ_max_ = 27.7°, θ_min_ = 2.4°
Absorption correction: multi-scan (*SADABS*; Sheldrick, 1996)	*h* = −23→23
*T*_min_ = 0.71, *T*_max_ = 0.86	*k* = −12→12
29673 measured reflections	*l* = −28→30

### Refinement

**Table:**

Refinement on *F*^2^	Primary atom site location: structure-invariant direct methods
Least-squares matrix: full	Secondary atom site location: difference Fourier map
*R*[*F*^2^ > 2σ(*F*^2^)] = 0.027	Hydrogen site location: inferred from neighbouring sites
*wR*(*F*^2^) = 0.076	H atoms treated by a mixture of independent and constrained refinement
*S* = 1.03	*w* = 1/[σ^2^(*F*_o_^2^) + (0.0415*P*)^2^ + 2.4079*P*] where *P* = (*F*_o_^2^ + 2*F*_c_^2^)/3
4625 reflections	(Δ/σ)_max_ = 0.002
259 parameters	Δρ_max_ = 0.28 e Å^−^^3^
5 restraints	Δρ_min_ = −0.27 e Å^−^^3^

### Special details

**Table:**

Geometry. All e.s.d.'s (except the e.s.d. in the dihedral angle between two l.s. planes) are estimated using the full covariance matrix. The cell e.s.d.'s are taken into account individually in the estimation of e.s.d.'s in distances, angles and torsion angles; correlations between e.s.d.'s in cell parameters are only used when they are defined by crystal symmetry. An approximate (isotropic) treatment of cell e.s.d.'s is used for estimating e.s.d.'s involving l.s. planes.
Refinement. Refinement of *F*^2^ against ALL reflections. The weighted *R*-factor *wR* and goodness of fit *S* are based on *F*^2^, conventional *R*-factors *R* are based on *F*, with *F* set to zero for negative *F*^2^. The threshold expression of *F*^2^ > σ(*F*^2^) is used only for calculating *R*-factors(gt) *etc*. and is not relevant to the choice of reflections for refinement. *R*-factors based on *F*^2^ are statistically about twice as large as those based on *F*, and *R*- factors based on ALL data will be even larger.

### Fractional atomic coordinates and isotropic or equivalent isotropic displacement parameters (Å^2^)

**Table:**

	*x*	*y*	*z*	*U*_iso_*/*U*_eq_	
Cu1	0.124473 (10)	0.670966 (18)	0.464441 (8)	0.03052 (7)	
C1	0.33281 (9)	0.85492 (16)	0.40975 (7)	0.0341 (3)	
C2	0.31963 (10)	0.94374 (17)	0.35537 (7)	0.0395 (4)	
C3	0.38265 (12)	1.0098 (2)	0.34354 (9)	0.0543 (5)	
H3A	0.4291	1.0076	0.3714	0.065*	
C4	0.37868 (14)	1.0808 (2)	0.29013 (11)	0.0684 (6)	
H4A	0.4219	1.1266	0.2834	0.082*	
C5	0.31253 (14)	1.0826 (2)	0.24877 (10)	0.0627 (6)	
H5A	0.3110	1.1271	0.2130	0.075*	
C6	0.24541 (13)	1.01821 (18)	0.25878 (8)	0.0485 (4)	
C7	0.24722 (10)	0.95047 (16)	0.31396 (7)	0.0381 (3)	
C8	0.17617 (14)	1.0204 (2)	0.21466 (8)	0.0568 (5)	
H8A	0.1752	1.0654	0.1791	0.068*	
C9	0.11186 (13)	0.9584 (2)	0.22331 (8)	0.0571 (5)	
H9A	0.0680	0.9554	0.1929	0.069*	
C10	0.11117 (11)	0.89818 (19)	0.27848 (8)	0.0460 (4)	
H10A	0.0660	0.8583	0.2846	0.055*	
C11	0.17582 (10)	0.89708 (16)	0.32346 (7)	0.0367 (3)	
C12	0.16459 (9)	0.85435 (16)	0.38382 (7)	0.0334 (3)	
C13	0.09853 (12)	0.4309 (2)	0.38365 (9)	0.0543 (5)	
H13A	0.1348	0.4846	0.3707	0.065*	
C14	0.1278 (3)	0.2623 (3)	0.31590 (17)	0.1263 (15)	
H14A	0.1622	0.3321	0.3085	0.189*	
H14B	0.0923	0.2401	0.2796	0.189*	
H14C	0.1572	0.1836	0.3310	0.189*	
C15	0.02931 (19)	0.2191 (3)	0.37687 (13)	0.0902 (9)	
H15A	0.0055	0.2632	0.4055	0.135*	
H15B	0.0551	0.1388	0.3940	0.135*	
H15C	−0.0096	0.1955	0.3426	0.135*	
C16	−0.03349 (10)	0.72571 (17)	0.46612 (8)	0.0418 (4)	
H16A	−0.0370	0.6338	0.4735	0.050*	
C17	−0.16911 (14)	0.7426 (3)	0.46719 (17)	0.0956 (10)	
H17A	−0.1636	0.6470	0.4735	0.143*	
H17B	−0.2080	0.7597	0.4320	0.143*	
H17C	−0.1841	0.7838	0.5007	0.143*	
C18	−0.09461 (13)	0.9433 (2)	0.44989 (12)	0.0661 (6)	
H18A	−0.0439	0.9698	0.4457	0.099*	
H18B	−0.1075	0.9902	0.4829	0.099*	
H18C	−0.1315	0.9656	0.4143	0.099*	
O1W	0.22801 (6)	0.59540 (12)	0.46744 (5)	0.0358 (2)	
H1WA	0.2520 (12)	0.641 (2)	0.4477 (8)	0.054*	
H1WB	0.2520 (12)	0.589 (2)	0.5009 (7)	0.054*	
O1	0.06739 (7)	0.47781 (13)	0.42143 (6)	0.0511 (3)	
O2	0.02961 (7)	0.77247 (12)	0.46251 (6)	0.0434 (3)	
O3	0.13036 (6)	0.74302 (12)	0.38655 (5)	0.0389 (2)	
O4	0.18619 (7)	0.93436 (12)	0.42557 (5)	0.0407 (3)	
O5	0.30792 (7)	0.73769 (11)	0.40428 (5)	0.0389 (3)	
O6	0.37233 (6)	0.90670 (12)	0.45727 (5)	0.0395 (3)	
N1	0.08503 (14)	0.30962 (18)	0.35940 (9)	0.0687 (5)	
N2	−0.09595 (8)	0.79924 (16)	0.46006 (7)	0.0458 (3)	

### Atomic displacement parameters (Å^2^)

**Table:**

	*U*^11^	*U*^22^	*U*^33^	*U*^12^	*U*^13^	*U*^23^
Cu1	0.02809 (11)	0.02933 (11)	0.03456 (11)	0.00029 (7)	0.00758 (7)	−0.00087 (7)
C1	0.0308 (7)	0.0369 (8)	0.0381 (8)	0.0040 (6)	0.0154 (6)	0.0037 (6)
C2	0.0456 (9)	0.0357 (8)	0.0405 (8)	0.0005 (7)	0.0167 (7)	0.0047 (7)
C3	0.0534 (11)	0.0562 (12)	0.0563 (11)	−0.0080 (9)	0.0181 (9)	0.0110 (9)
C4	0.0724 (15)	0.0669 (14)	0.0723 (15)	−0.0164 (12)	0.0299 (12)	0.0220 (12)
C5	0.0888 (16)	0.0545 (12)	0.0502 (11)	−0.0057 (11)	0.0271 (11)	0.0172 (9)
C6	0.0709 (12)	0.0388 (9)	0.0386 (9)	0.0042 (8)	0.0179 (8)	0.0068 (7)
C7	0.0525 (10)	0.0301 (7)	0.0338 (8)	0.0041 (7)	0.0137 (7)	0.0039 (6)
C8	0.0837 (15)	0.0503 (11)	0.0345 (9)	0.0080 (10)	0.0086 (9)	0.0115 (8)
C9	0.0696 (13)	0.0567 (11)	0.0391 (10)	0.0086 (10)	−0.0020 (9)	0.0083 (9)
C10	0.0512 (10)	0.0445 (10)	0.0388 (9)	0.0060 (8)	0.0018 (7)	0.0035 (7)
C11	0.0460 (9)	0.0296 (8)	0.0339 (8)	0.0074 (6)	0.0075 (7)	0.0016 (6)
C12	0.0294 (7)	0.0356 (7)	0.0348 (8)	0.0099 (5)	0.0057 (6)	0.0046 (6)
C13	0.0593 (12)	0.0406 (10)	0.0641 (12)	−0.0089 (9)	0.0157 (10)	−0.0125 (9)
C14	0.204 (4)	0.0723 (19)	0.129 (3)	−0.004 (2)	0.093 (3)	−0.0396 (19)
C15	0.120 (2)	0.0554 (14)	0.094 (2)	−0.0337 (16)	0.0198 (17)	−0.0182 (14)
C16	0.0412 (9)	0.0345 (8)	0.0528 (10)	0.0041 (7)	0.0171 (8)	0.0013 (7)
C17	0.0436 (12)	0.0725 (17)	0.180 (3)	0.0018 (12)	0.0436 (16)	0.0084 (19)
C18	0.0562 (12)	0.0503 (12)	0.0948 (17)	0.0194 (10)	0.0228 (12)	0.0182 (11)
O1W	0.0315 (5)	0.0401 (6)	0.0366 (6)	0.0036 (4)	0.0088 (4)	0.0051 (5)
O1	0.0484 (7)	0.0433 (7)	0.0632 (8)	−0.0096 (6)	0.0151 (6)	−0.0163 (6)
O2	0.0338 (6)	0.0384 (6)	0.0601 (8)	0.0058 (5)	0.0147 (5)	0.0016 (6)
O3	0.0368 (6)	0.0414 (6)	0.0378 (6)	0.0003 (5)	0.0067 (5)	0.0057 (5)
O4	0.0430 (6)	0.0438 (6)	0.0348 (6)	0.0076 (5)	0.0069 (5)	−0.0017 (5)
O5	0.0431 (6)	0.0337 (6)	0.0427 (6)	0.0024 (5)	0.0156 (5)	0.0040 (5)
O6	0.0365 (6)	0.0442 (6)	0.0388 (6)	−0.0036 (5)	0.0103 (5)	0.0041 (5)
N1	0.0966 (15)	0.0409 (9)	0.0714 (12)	−0.0079 (9)	0.0241 (11)	−0.0189 (8)
N2	0.0344 (7)	0.0431 (8)	0.0626 (10)	0.0063 (6)	0.0166 (7)	0.0011 (7)

### Geometric parameters (Å, °)

**Table:**

Cu1—O2	1.9500 (11)	C12—O3	1.266 (2)
Cu1—O6^i^	1.9505 (11)	C13—O1	1.217 (2)
Cu1—O3	1.9544 (11)	C13—N1	1.324 (2)
Cu1—O1W	1.9678 (11)	C13—H13A	0.9300
Cu1—O1	2.2885 (12)	C14—N1	1.456 (3)
C1—O5	1.2386 (19)	C14—H14A	0.9600
C1—O6	1.275 (2)	C14—H14B	0.9600
C1—C2	1.508 (2)	C14—H14C	0.9600
C2—C3	1.370 (2)	C15—N1	1.452 (3)
C2—C7	1.428 (2)	C15—H15A	0.9600
C3—C4	1.406 (3)	C15—H15B	0.9600
C3—H3A	0.9300	C15—H15C	0.9600
C4—C5	1.344 (3)	C16—O2	1.228 (2)
C4—H4A	0.9300	C16—N2	1.307 (2)
C5—C6	1.410 (3)	C16—H16A	0.9300
C5—H5A	0.9300	C17—N2	1.453 (3)
C6—C8	1.417 (3)	C17—H17A	0.9600
C6—C7	1.432 (2)	C17—H17B	0.9600
C7—C11	1.430 (2)	C17—H17C	0.9600
C8—C9	1.345 (3)	C18—N2	1.447 (3)
C8—H8A	0.9300	C18—H18A	0.9600
C9—C10	1.406 (3)	C18—H18B	0.9600
C9—H9A	0.9300	C18—H18C	0.9600
C10—C11	1.371 (2)	O1W—H1WA	0.82 (2)
C10—H10A	0.9300	O1W—H1WB	0.80 (1)
C11—C12	1.507 (2)	O6—Cu1^i^	1.9505 (11)
C12—O4	1.242 (2)		
			
O2—Cu1—O6^i^	94.60 (5)	O3—C12—C11	116.67 (14)
O2—Cu1—O3	90.25 (5)	O1—C13—N1	125.4 (2)
O6^i^—Cu1—O3	175.05 (5)	O1—C13—H13A	117.3
O2—Cu1—O1W	171.31 (5)	N1—C13—H13A	117.3
O6^i^—Cu1—O1W	88.48 (5)	N1—C14—H14A	109.5
O3—Cu1—O1W	86.58 (5)	N1—C14—H14B	109.5
O2—Cu1—O1	96.99 (5)	H14A—C14—H14B	109.5
O6^i^—Cu1—O1	89.67 (5)	N1—C14—H14C	109.5
O3—Cu1—O1	90.71 (5)	H14A—C14—H14C	109.5
O1W—Cu1—O1	91.15 (5)	H14B—C14—H14C	109.5
O5—C1—O6	125.65 (15)	N1—C15—H15A	109.5
O5—C1—C2	118.24 (15)	N1—C15—H15B	109.5
O6—C1—C2	116.03 (14)	H15A—C15—H15B	109.5
C3—C2—C7	119.91 (16)	N1—C15—H15C	109.5
C3—C2—C1	117.06 (16)	H15A—C15—H15C	109.5
C7—C2—C1	122.75 (14)	H15B—C15—H15C	109.5
C2—C3—C4	121.4 (2)	O2—C16—N2	122.97 (16)
C2—C3—H3A	119.3	O2—C16—H16A	118.5
C4—C3—H3A	119.3	N2—C16—H16A	118.5
C5—C4—C3	120.1 (2)	N2—C17—H17A	109.5
C5—C4—H4A	120.0	N2—C17—H17B	109.5
C3—C4—H4A	120.0	H17A—C17—H17B	109.5
C4—C5—C6	121.06 (18)	N2—C17—H17C	109.5
C4—C5—H5A	119.5	H17A—C17—H17C	109.5
C6—C5—H5A	119.5	H17B—C17—H17C	109.5
C5—C6—C8	120.45 (17)	N2—C18—H18A	109.5
C5—C6—C7	119.75 (19)	N2—C18—H18B	109.5
C8—C6—C7	119.79 (18)	H18A—C18—H18B	109.5
C2—C7—C11	125.34 (14)	N2—C18—H18C	109.5
C2—C7—C6	117.57 (16)	H18A—C18—H18C	109.5
C11—C7—C6	117.08 (16)	H18B—C18—H18C	109.5
C9—C8—C6	121.18 (17)	Cu1—O1W—H1WA	110.9 (15)
C9—C8—H8A	119.4	Cu1—O1W—H1WB	111.5 (16)
C6—C8—H8A	119.4	H1WA—O1W—H1WB	110.4 (19)
C8—C9—C10	119.82 (18)	C13—O1—Cu1	113.79 (12)
C8—C9—H9A	120.1	C16—O2—Cu1	126.56 (11)
C10—C9—H9A	120.1	C12—O3—Cu1	118.88 (10)
C11—C10—C9	121.36 (19)	C1—O6—Cu1^i^	122.57 (10)
C11—C10—H10A	119.3	C13—N1—C15	121.0 (2)
C9—C10—H10A	119.3	C13—N1—C14	120.5 (2)
C10—C11—C7	120.33 (15)	C15—N1—C14	118.4 (2)
C10—C11—C12	116.57 (15)	C16—N2—C18	121.61 (16)
C7—C11—C12	122.64 (14)	C16—N2—C17	121.86 (18)
O4—C12—O3	126.08 (15)	C18—N2—C17	116.40 (17)
O4—C12—C11	117.15 (14)		
			
O5—C1—C2—C3	−129.54 (18)	C2—C7—C11—C12	14.1 (2)
O6—C1—C2—C3	47.3 (2)	C6—C7—C11—C12	−164.70 (15)
O5—C1—C2—C7	44.3 (2)	C10—C11—C12—O4	−124.12 (16)
O6—C1—C2—C7	−138.81 (16)	C7—C11—C12—O4	48.1 (2)
C7—C2—C3—C4	−2.4 (3)	C10—C11—C12—O3	52.4 (2)
C1—C2—C3—C4	171.7 (2)	C7—C11—C12—O3	−135.41 (15)
C2—C3—C4—C5	−1.4 (4)	N1—C13—O1—Cu1	168.26 (19)
C3—C4—C5—C6	2.4 (4)	O2—Cu1—O1—C13	135.72 (15)
C4—C5—C6—C8	−179.7 (2)	O6^i^—Cu1—O1—C13	−129.68 (15)
C4—C5—C6—C7	0.5 (3)	O3—Cu1—O1—C13	45.38 (15)
C3—C2—C7—C11	−173.81 (17)	O1W—Cu1—O1—C13	−41.21 (15)
C1—C2—C7—C11	12.5 (3)	N2—C16—O2—Cu1	−174.76 (13)
C3—C2—C7—C6	5.0 (2)	O6^i^—Cu1—O2—C16	−59.16 (15)
C1—C2—C7—C6	−168.68 (15)	O3—Cu1—O2—C16	121.82 (15)
C5—C6—C7—C2	−4.1 (3)	O1—Cu1—O2—C16	31.08 (15)
C8—C6—C7—C2	176.05 (17)	O4—C12—O3—Cu1	−10.9 (2)
C5—C6—C7—C11	174.82 (17)	C11—C12—O3—Cu1	172.91 (10)
C8—C6—C7—C11	−5.0 (2)	O2—Cu1—O3—C12	87.85 (11)
C5—C6—C8—C9	179.4 (2)	O1W—Cu1—O3—C12	−84.05 (11)
C7—C6—C8—C9	−0.8 (3)	O1—Cu1—O3—C12	−175.16 (11)
C6—C8—C9—C10	4.5 (3)	O5—C1—O6—Cu1^i^	−18.5 (2)
C8—C9—C10—C11	−2.2 (3)	C2—C1—O6—Cu1^i^	164.90 (10)
C9—C10—C11—C7	−3.8 (3)	O1—C13—N1—C15	0.0 (4)
C9—C10—C11—C12	168.60 (17)	O1—C13—N1—C14	−177.9 (3)
C2—C7—C11—C10	−173.94 (16)	O2—C16—N2—C18	−1.4 (3)
C6—C7—C11—C10	7.2 (2)	O2—C16—N2—C17	−177.2 (2)

Symmetry codes: (i) −*x*+1/2, −*y*+3/2, −*z*+1.

### Hydrogen-bond geometry (Å, °)

**Table:**

*D*—H···*A*	*D*—H	H···*A*	*D*···*A*	*D*—H···*A*
O1W—H1WA···O5	0.82 (2)	1.82 (2)	2.642 (2)	178 (2)
O1W—H1WB···O4^i^	0.80 (1)	1.82 (2)	2.623 (2)	175 (2)
C3—H3A···O1^ii^	0.93	2.49	3.396 (3)	164
C13—H13A···O3	0.93	2.59	3.140 (2)	119
C17—H17A···O6^iii^	0.96	2.51	3.424 (3)	159

Symmetry codes: (i) −*x*+1/2, −*y*+3/2, −*z*+1; (ii) *x*+1/2, *y*+1/2, *z*; (iii) *x*−1/2, *y*−1/2, *z*.
